# Alterations in Cortical-Subcortical Metabolism in Temporal Lobe Epilepsy With Impaired Awareness Seizures

**DOI:** 10.3389/fnagi.2022.849774

**Published:** 2022-03-10

**Authors:** Jiale Hou, Haoyue Zhu, Ling Xiao, Charlie Weige Zhao, Guang Liao, Yongxiang Tang, Li Feng

**Affiliations:** ^1^Department of Nuclear Medicine, Xiangya Hospital, Central South University, Changsha, China; ^2^Department of Neurology, Xiangya Hospital, Central South University, Changsha, China; ^3^Yale University School of Medicine, New Haven, CT, United States; ^4^National Clinical Research Center for Geriatric Disorders (XIANGYA), Xiangya Hospital, Central South University, Changsha, China

**Keywords:** metabolism, FDG-PET, temporal lobe epilepsy, impaired awareness seizures, cortical-subcortical network

## Abstract

**Objective:**

The features of cerebral metabolism associated with loss of consciousness in patients with temporal lobe epilepsy (TLE) have not been fully elucidated. We aim to investigate the alterations in cortical-subcortical metabolism in temporal lobe epilepsy with impaired awareness seizures (IAS).

**Methods:**

Regional cerebral metabolism was measured using fluorine-18-fluorodeoxyglucose positron emission tomography (^18^F-FDG PET) in patients with TLE-IAS and healthy controls. All patients had a comprehensive evaluation to confirm their seizure origin and lateralization. Videos of all seizures were viewed and rated by at least two epileptologists to identify the state of consciousness when a seizure occurred. By synthesizing the seizure history, semeiology, and video EEG of all patients, as long as the patients had one seizure with impaired awareness, she/he will be included. 76 patients with TLE-IAS and 60 age-matched healthy controls were enrolled in this study. Regional cerebral metabolic patterns were analyzed for TLE-IAS and healthy control groups using statistical parametric mapping. Besides, we compared the MRI-negative patients and MRI-positive patients with healthy controls, respectively.

**Results:**

There were no significant differences in the age and sex of TLE-IAS patients and healthy control. TLE-IAS patients showed extensive bilateral hypermetabolism in the frontoparietal regions, cingulate gyrus, corpus callosum, occipital lobes, basal ganglia, thalamus, brainstem, and cerebellum. The region of metabolic change was more extensive in right TLE-IAS than that of the left, including extensive hypometabolism in the ipsilateral temporal, frontal, parietal, and insular lobes. And contralateral temporal lobe, bilateral frontoparietal regions, occipital lobes, the anterior and posterior regions of the cingulate gyrus, bilateral thalamus, bilateral basal ganglia, brainstem, and bilateral cerebellum showed hypermetabolism. The TLE patients with impaired awareness seizure showed hypermetabolism in the cortical-subcortical network including the arousal system. Additionally, 48 MRI-positive and 28 MRI-negative TLE-IAS patients were included in our study. TLE-IAS patients with MRI-negative and MRI-positive were both showed hypermetabolism in the cingulate gyrus. Hypometabolism in the bilateral temporal lobe was showed in the TLE-IAS with MRI-positive.

**Conclusion:**

These findings suggested that the repetitive consciousness impairing ictal events may have an accumulative effect on brain metabolism, resulting in abnormal interictal cortical-subcortical metabolic disturbance in TLE patients with impaired awareness seizure. Understanding these metabolic mechanisms may guide future clinical treatments to prevent seizure-related awareness deficits and improve quality of life in people with TLE.

## Introduction

Epilepsy results in a wide range of deficits, including cognitive, behavioral, psychiatric, and other neurologic comorbidities (Devinsky et al., 2018). Seizure-related loss of consciousness is a major contributor to the morbidity and mortality of epilepsy, severely affecting the patient’s safety, productivity, emotional health (Zijlmans et al., 2019). Temporal lobe epilepsy (TLE) is the most common epilepsy of adulthood (Jones and Cascino, 2016). Although seizures of TLE arose from focal lobes, it can affect remoted regions, resulting in an overall level of impaired consciousness/responsiveness, which was classified as focal impaired awareness seizures (FIAS) and it has a higher prevalence than focal aware seizures and an enormous impact on patient quality of life (Berg, 2008; Blumenfeld and Meador, 2014; Fisher et al., 2017a).

Emerging evidence from behavioral, electrophysiological, and neuroimaging experiments from both human and animal models suggests that awareness impairment in TLE can occur either through direct seizure involvement of cortical-subcortical structures or *via* indirect network inhibition (Li et al., 2019; Xu et al., 2021), both of which ultimately result in cortical suppression (Griffin and Speck, 2004; Calabrò et al., 2015; Koch et al., 2016; Gutfreund, 2017). In TLE patients with impaired awareness seizures (IAS) and rodent models of limbic seizures, our group and other collaborators previously demonstrated network alterations between cortical and subcortical structures important for consciousness regulation (Guye et al., 2006; Arthuis et al., 2009), and have found relationships between these network changes and neuropsychological deficits (Blumenfeld et al., 2004b,^2009^; Blumenfeld, 2012; Feng et al., 2017). Because the majority of IAS studies focus on brain structures abnormalities or functional alteration including functional magnetic resonance imaging (fMRI) and electrophysiology, the role of metabolic networks involved in IAS and the differences of metabolic mechanism in TLE patients with IAS has been underexamined despite indications of its potential importance.

Positron emission tomography (PET), the most sensitive method of imaging trace molecules *in vivo*, is a useful tool to assess cerebral metabolism and can measure biochemical and physiological processes in the whole brain with three-dimensional resolution (Duncan et al., 2016). PET studies in patients with disorders of consciousness reveal alterations in a large-scale brain network encompassing the polymodal associative cortices, where the core pathology is related to an abnormality of brain function rather than macroscopic structures (Laureys et al., 1999; Di Perri et al., 2014). Thus, by comparing PET of TLE-IAS patients to healthy controls, we can investigate the underlying metabolic changes associated with seizure-related loss of consciousness (Nakayama et al., 2006).

We conducted a fluorine-18-fluorodeoxyglucose PET (^18^F-FDG PET) study analyzed using statistical parametric mapping (SPM) in TLE patients with IAS. SPM is a voxel-by-voxel analysis method that avoids subjectivity during data interpretation (Chassoux et al., 2017), addressing one of the key limitations of the region of interest (ROI)-based techniques (Tan et al., 2019). We hypothesized that ictal events in TLE patients with IAS lead to persistent interictal cortical-subcortical network disturbances that may affect both neocortical connectivity and neurocognitive function. Long-term disturbances between cortical-subcortical systems affect regional cerebral metabolism, contributing to impaired consciousness and cognition.

## Materials and Methods

### Study Participants

According to International League Against Epilepsy (ILAE) 2017 classification of seizure types (Fisher, 2017; Fisher et al., 2017a,b), 76 patients with TLE-IAS who underwent evaluation for epilepsy surgery at Xiangya Hospital, Central South University from January 1, 2016, to August 31, 2019, were recruited and consented. These patients had no history of head injury, cerebral stroke, intracranial operation, psychiatric illness, or substance use disorder. In addition, a total of 60 healthy controls matched for sex, age, and education were randomly recruited in the current study. For all enrolled participants, we carried out clinical and neuropsychological testing, assessed their state of consciousness during seizures, and performed FDG-PET studies, protocols detailed below. Institutional Review Board approvals and informed consents were obtained prior to all procedures. For minors, consent was obtained from their parents.

### Clinical and Consciousness Assessments

All patients had a comprehensive evaluation based on findings on detailed seizure history, semiology, neurological examination, neuropsychological testing, neuroimaging, surface or video electroencephalography (EEG) recording, or invasive stereo-EEG (SEEG) monitoring. The seizure origin and laterality location were defined by a multidisciplinary team and further partly confirmed by operative neuropathology and a following-up study. Videos of all seizures were viewed and rated by at least two epileptologists at our institution to identify the state of consciousness of the patient during seizures. The patients were presented with questions or commands during seizures by either medical staff or family members. Any impaired response to verbal questions, failure to follow simple commands, or amnesiac events during the seizure was classified as impaired awareness, whereas patients who remained fully alert and appropriately interactive during a seizure were classified as having retained awareness and excluded from our study (Blumenfeld et al., 2004b; Blumenfeld, 2012; Fisher, 2017; Fisher et al., 2017a,b). By synthesizing the seizure history, semeiology, and video EEG of all patients, as long as the patient had one seizure with impaired awareness, she/he will be included.

### Fluorine-18-Fluorodeoxyglucose Positron Emission Tomography Image Examination

Fluorine-18-fluorodeoxyglucose positron emission tomography was performed at the PET Center of Xiangya Hospital using a Discovery Elite PET/CT scanner (GE Healthcare, Boston, MA, United States) within one week of clinical assessment. Participants discontinued all antiseizure drugs (ASDs) for at least 24 h and fasted for at least 6 h before injection of FDG. The patient has no clinically visible seizures within 24 h before ^18^F-FDG PET examination and would have a continuous EEG recording to ensure that there was no seizure 2 h before FDG injection, monitoring started before injection (2 h before) to ensure that FDG is not administered in a postictal situation (Varrone et al., 2009). A dose of 3.7 MBq/kg of FDG was injected intravenously through the cubital vein over a period of one minute. Then a static three-dimensional PET image was collected in about 60 min. Participants were placed in the PET scanner so that slices were parallel to the canthomeatal line. The full width of the scan at half maximum was 5.4 mm. All images were reconstructed as a 256 × 256 trans-axial matrix (35 cm field of view) using the 3D VUE Point (GE Healthcare) ordered-subset expectation-maximization algorithm with six iterations and six subsets, which produced 47 transaxial images at 3.25 mm intervals. A low dose CT scan was obtained simultaneously for attenuation correction (Tang et al., 2018).

### Data Analysis

Image processing was performed using the SPM (Wellcome Department of Cognitive Neurology, London, United Kingdom) implemented on MATLAB. Individual ^18^F-FDG PET image volumes were spatially normalized into standard stereotactic Montreal Neurological Institute (MNI) space with voxel sizes of 2 × 2 × 2. An 8-mm full-width-half-maximum Gaussian kernel was used to improve between-participant spatial alignment and smooth data for statistical analysis.

Once the images were spatially normalized and smoothed, a general linear model was used to carry out the appropriate voxel-by-voxel univariate statistical tests. Image intensity between participants was normalized to prevent interparticipant variability in cerebral tracer uptake from masking regional changes. This was done using proportional scaling, which scales each image proportionally to the mean global brain activity. The analysis produced a t-statistic for each voxel, as specified by the contrast, which constituted the statistical parametric map SPM{T}. The SPM{T} map was then transformed to the unit normal distribution to give a gaussian field or SPM{Z}.

Metabolic changes were considered statistically significant when family wise error (FWE) or false discovery rate (FDR) corrected *p* < 0.05 with cluster size (k_E_) above 20 contiguous voxels. After data preprocessing using SPM, significant clusters were visualized, reported, and anatomically labeled using the xjView Matlab toolbox.^1^ Data of metabolic profile information about the clusters were obtained, including the number of voxels, anatomical location (in terms of MNI coordinates), and peak intensity of each cluster.

### Statistical Analyses

Clinical data analyses were performed with statistical software. Descriptive statistics were summarized as mean ± SD or median and interquartile range. Comparisons between groups were made with the Student’s *t*-test or Mann-Whitney *U* test for quantitative variables and with the chi-square test or Fisher’s test for qualitative variables. All statistical tests were two-sided, and *p* < 0.05 indicated statistical significance. The statistical analyses were performed using SPSS software for Windows (IBM SPSS Statistics, Version 18.0).

For PET image analysis, we first compared baseline glucose uptake values of the TLE-IAS group (Total group, then left TLE-IAS and right TLE-IAS) and healthy controls using analysis of covariance (ANCOVA) with the group as the between-subject factor and age and sex as confounding covariates. And we also compared the MRI-negative and MRI-positive TLE patients with healthy controls using the same way, respectively. A two-sample *t*-test was used to compare the different groups. Metabolic changes in the whole brain and cerebellum were then calculated, comparing the TLE-IAS group to healthy control. Finally, the spatial coordinates of the areas of metabolic change were identified in the whole brain and cerebellum in the two groups, using an atlas to identify brain regions (Automated Anatomical Labeling areas, AAL) and to approximate Brodmann areas.

## Results

### Clinical Data

After screening, 76 TLE patients (42 male and 34 female; mean age 26.0 ± 10.2 years) were included. 60 volunteers (32 male and 28 female; mean age 25.7 ± 10.0 years) were recruited as healthy controls. Thereinto, 12 patients underwent SEEG monitoring. 48 patients underwent an operation for intractable epilepsy with a pathologic diagnosis. All patients enrolled were unilateral TLE. The clinical data were shown in Table 1. There was no significant difference between the two groups concerning age or sex (Table 1).

**TABLE 1:** Demographics of patients and controls.

	TLE-IAS	Control	Significance
Age (years)	26.0 (10.2)	25.7 (10.0)	0.111
Age range (years)	6-55	5-48	–
Sex (male: female)	42:34	32:28	0.982
Onset age of seizure (years)	14.8 (9.7)	–	–
Duration of seizure (years)	11.0 (7.6)	–	–
SEEG	12	–	–
**Postoperative pathology**			
FCD	10	–	–
HS	19	–	–
Tumor	2		
Other	17	–	–
**Epileptogenic zone**			
Left temporal lobe	41	–	–
Right temporal lobe	35	–	–

*Values are presented as mean (SD). FCD, focal cortical dysplasia; TLE-IAS, temporal lobe epilepsy with impaired awareness seizures; HS, hippocampal sclerosis; NS, not significant; SEEG, stereo-electroencephalography.*

### Metabolic Abnormalities in Patients With Temporal Lobe Epilepsy-Impaired Awareness Seizures Versus Healthy Controls

**FIGURE 1:**
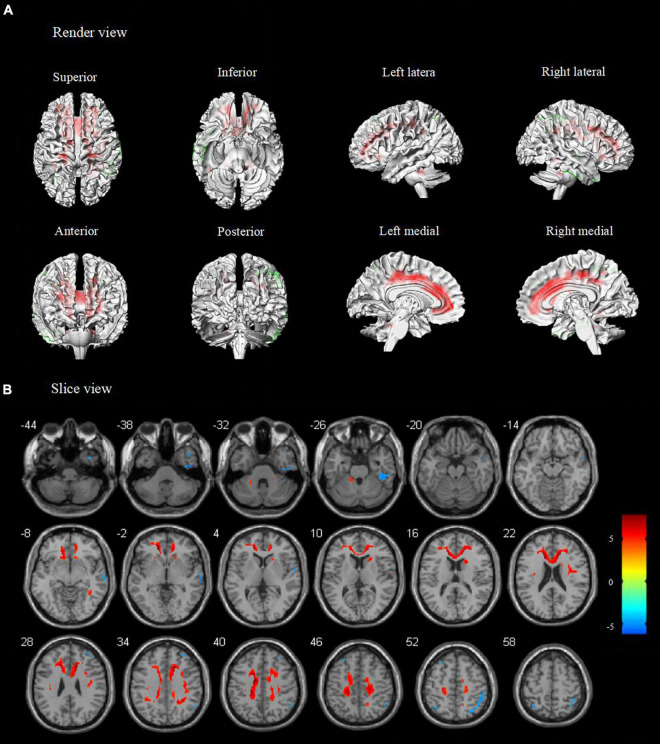
Metabolic features in temporal lobe epilepsy with impaired awareness seizures (FWE corrected). Comparison of temporal lobe epilepsy with awareness impairing seizures (*N* = 76) versus healthy controls (*N* = 60) was performed in **(A)** render view and **(B)** slice view (FWE corrected *p* < 0.05 with cluster thresholding, *k* = 20 voxels). Voxels with significantly low uptake are shown in green and voxels with significantly high uptake are shown in red.

**FIGURE 2:**
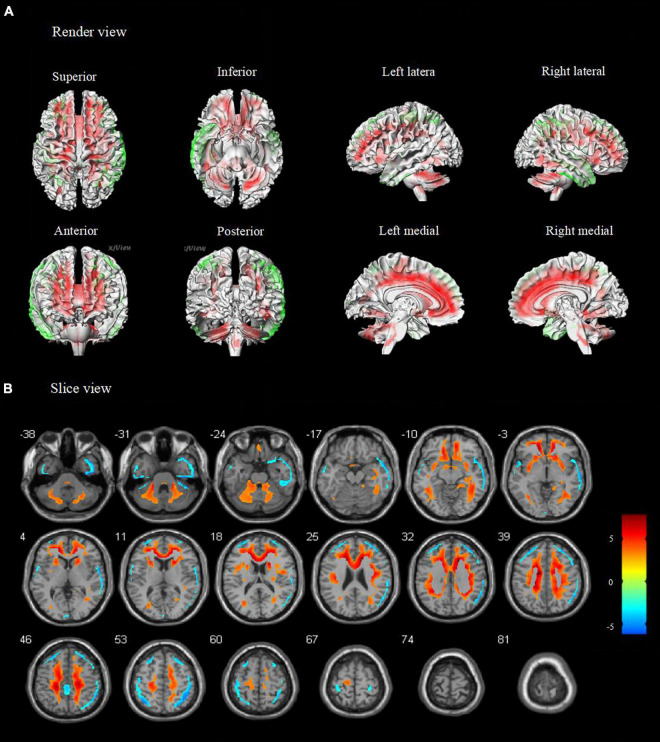
Metabolic features in temporal lobe epilepsy with impaired awareness seizures (FDR corrected). Comparison of temporal lobe epilepsy with awareness impairing seizures (*N* = 76) versus healthy controls (*N* = 60) was performed in **(A)** render view and **(B)** slice view (FDR corrected *p* < 0.05 with cluster thresholding, *k* = 20 voxels). Voxels with significantly low uptake are shown in green and voxels with significantly high uptake are shown in red.

Compared to healthy controls, patients with TLE-IAS had hypermetabolism in the bilateral frontoparietal regions, the anterior and posterior regions of the cingulate gyrus, bilateral cerebellum (*p* < 0.05, FWE corrected), and bilateral occipital lobes, thalamus, basal ganglia, corpus callosum, brainstem (*p* < 0.05, FDR corrected). Due to the mixed laterality of TLE patients, only small areas of hypometabolism were seen in the bilateral temporal lobe, frontal lobe, and parietal lobe (*p* < 0.05, FWE corrected). Interestingly, extensive hypermetabolism in the bilateral cerebellum was observed (Figures 1, 2).

**FIGURE 3:**
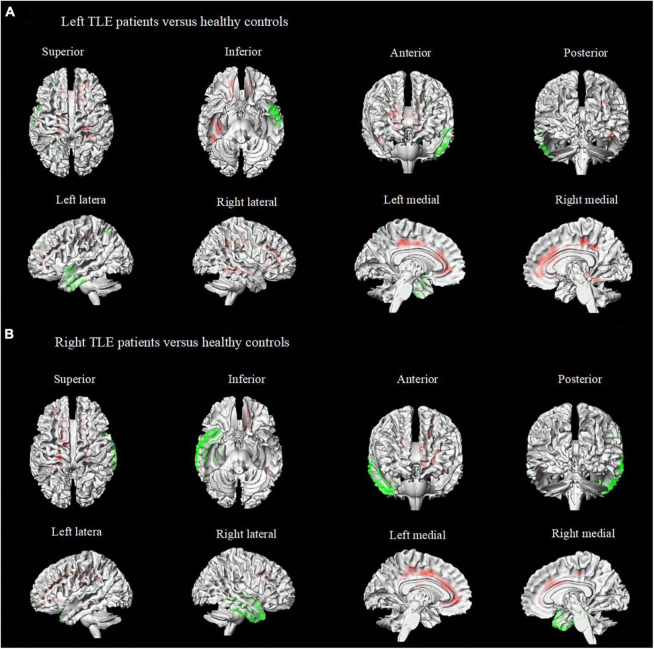
Metabolic features in patients with right and left temporal lobe epilepsy with impaired awareness seizures. Comparison of patients with differed in right and left TLE-IAS patients versus healthy controls (*N* = 60) (FWE corrected *p* < 0.05 with cluster thresholding, *k* = 20 voxels). Voxels with significantly low uptake are shown in green and voxels with significantly high uptake are shown in red. **(A)** Left TLE-IAS patients versus healthy controls, **(B)** Right TLE-IAS patients versus healthy controls. Patients from both laterality groups consistently displayed marked hypermetabolism in the contralateral temporal lobe, frontoparietal regions, the regions of the cingulate gyrus, along with hypometabolism in the ipsilateral temporal lobe, bilateral frontal lobe, insular lobe, and parietal lobe. The heat map depicts *t*-values. Coordinate and regional details are presented in Table 2. TLE-IAS, temporal lobe epilepsy with impaired awareness seizures.

To avoid data mixing from different lateralities of seizure onset and to examine if there were different metabolic patterns or metabolic networks due to seizure lateralization, we compared cerebral metabolism in the left and right TLE-IAS patients separately against healthy controls (Figure 3 and Table 2). In left TLE-IAS, patients had hypometabolism in the ipsilateral temporal, frontal, parietal, and insula cortices (*p* < 0.05, FWE corrected), and large areas of hypermetabolism in the contralateral temporal, bilateral frontoparietal regions, and anterior cingulate gyrus (*p* < 0.05, FWE corrected) and bilateral occipital lobes, brainstem, thalamus, basal ganglia, and bilateral cerebellum (*p* < 0.05, FDR corrected). The region of metabolic change was more extensive in right TLE-IAS than that of the left, including extensive hypometabolism in the ipsilateral temporal, parietal, and insular lobes (*p* < 0.05, FWE corrected) and hypermetabolism in the contralateral temporal lobe, bilateral frontoparietal regions, the anterior and posterior regions of the cingulate gyrus (*p* < 0.05, FWE corrected) and bilateral occipital lobes, brainstem, thalamus, basal ganglia, and bilateral cerebellum (*p* < 0.05, FDR corrected).

**TABLE 2:** Location and peaks of significant reduction/increasing in glucose metabolism in patients with TLE-IAS compared with normal controls.

Cluster-level k_E_	Voxel-level T	p _FWE–corrected_	Peak coordinates (x, y, z) (mm)			Anatomical region	Brodmann area
**TLE versus control**							
4456	7.83	0.000	16	20	30	Right cingulate gyrus	
	7.55	0.000	−14	−6	40	Left cingulate gyrus	
	7.31	0.000	−14	28	22	Left anterior cingulate	
57	6.09	0.000	−32	−44	30	Left parietal lobe	
31	5.46	0.002	16	−54	48	Right precuneus	
53	5.46	0.002	−14	−40	−28	Left cerebellum anterior lobe	
22	5.16	0.005	18	−36	−30	Right cerebellum anterior lobe	
36	4.96	0.011	38	−48	−6	Right temporal lobe	
179	−6.05 −5.96 −5.74	0.000 0.000 0.000	42 42 50	−22 −14 −14	−30 −38 −36	Right fusiform gyrus Right fusiform gyrus Left inferior temporal gyrus	
50	−5.43	0.002	−32	−62	56	Left superior parietal lobule	
47 25 225 71 27 49 26	−5.42 −5.21 −4.93 −5.32 −4.92 −5.28 −5.25 −5.06 −5.27 −5.21 −5.14 −4.68 −4.89 −4.68	0.002 0.004 0.012 0.003 0.013 0.003 0.004 0.008 0.003 0.004 0.006 0.032 0.015 0.031	42 46 40 58 52 48 40 28 66 62 32 42 −26 −30	12 14 4 4 10 −44 −50 −70 −16 −2 44 42 34 38	−40 −32 −44 −16 −24 54 56 52 −4 6 38 28 50 44	Right middle temporal lobule Right superior temporal gyrus Right middle temporal lobule Right middle temporal lobule Right superior temporal gyrus Right inferior parietal lobule Right inferior parietal lobule Right superior parietal lobule Right middle temporal lobule Right superior temporal gyrus Right superior frontal gyrus Right middle frontal gyrus Left middle frontal gyrus Left middle frontal gyrus	38 39 7 21 22 8
**Left TLE versus control**							
375	6.93	0.000	−20	−28	40	Left cingulate gyrus	
	6.14	0.000	−14	−6	40	Left cingulate gyrus	
	5.24	0.006	−16	6	40	Left cingulate gyrus	
389	6.32	0.000	16	20	30	Right cingulate gyrus	
	6.28	0.000	18	38	12	Right frontal lobe/sub-gyral	
	6.06	0.000	−16	28	24	Left anterior cingulate	
529	6.28 6.01 5.86	0.000 0.000 0.001	36 24 24	−42 −46 −26	32 34 34	Right parietal lobe/sub-gyral Right parietal lobe/sub-gyral Right frontal lobe/sub-gyral	
432	6.14	0.000	32	−28	−8	Right lateral ventricle/hippocampus	
	5.58 5.57	0.002 0.002	40 42	−6 −42	−18 −10	Right temporal lobe Right temporal lobe	
81	5.64	0.001	20	16	18	Right extra-nuclear	
	5.00	0.016	18	24	−4	Right frontal lobe/sub-gyral	
882 90 45 28 21	−6.83 −6.58 −6.52 −5.11 −4.91 −4.84 −4.72	0.000 0.000 0.000 0.003 0.007 0.009 0.015	−60 −40 −52 −34 46 −28 −26	−8 −18 8 −60 −46 60 46	−12 −32 −6 54 54 14 36	Left middle temporal gyrus Left fusiform gyrus Left Superior temporal gyrus Left superior parietal lobule Left inferior parietal lobule Left middle frontal gyrus Left superior frontal gyrus	7 10 9
**Right TLE versus control**							
1384	7.01	0.000	−14	−4	40	Left cingulate gyrus	
	6.78	0.000	−14	42	−2	Left anterior cingulate	
	6.61	0.000	−14	28	22	Left anterior cingulate	
312	6.64	0.000	18	22	32	Right cingulate gyrus	
	5.26	0.008	10	8	30	Right cingulate gyrus	
	5.16	0.011	18	38	12	Right frontal lobe/sub-gyral	
61	5.83	0.001	−32	−44	30	Left parietal lobe/sub-gyral	
77	5.70	0.001	20	−20	44	Right cingulate gyrus	24
50	5.48	0.003	30	6	30	Right frontal lobe/sub-gyral	
	5.25	0.008	26	4	38	Right frontal lobe/sub-gyral	
	5.10	0.013	34	0	24	Right frontal lobe/sub-gyral	
33	5.45	0.004	−40	−18	−12	Left temporal lobe/sub-gyral	
	4.88	0.030	−40	−6	−18	Left temporal lobe/sub-gyral	
65	5.36	0.005	−28	40	14	Left frontal lobe/sub-gyral	
24	5.21 5.31	0.009 0.006	−26 −44	48 −42	2 −10	Left frontal lobe/sub-gyral Left temporal lobe/sub-gyral	
2121	−8.76	0.000	46	14	−32	Right superior temporal gyrus	38
	−8.35	0.000	56	6	−20	Right middle temporal gyrus	
	−8.30	0.000	42	−8	−42	Right inferior temporal gyrus	
45	−5.15	0.011	58	−54	32	Right supramarginal gyrus	40
	−5.03	0.018	54	−60	36	Right angular gyrus	

*TLE-IAS, temporal lobe epilepsy with impaired awareness seizures; k_E_, cluster size; FEW, family wise error.*

### Metabolic Changes in Temporal Lobe Epilepsy-Impaired Awareness Seizures Patients With MRI-Negative and MRI-Positive

In our study, we had 28 patients with MRI-negative and 48 patients with MRI-positive. TLE-IAS patients with MRI-positive were older than that MRI-negative (27.8 ± 10.6 versus 22.8 ± 8.6). However, the age difference between the two groups was not clinically significant. And there was no significant difference in gender, age of onset, and duration time between the two groups (Supplementary Table 1). Then, we compared MRI-negative and MRI-positive TLE-IAS patients with healthy controls, respectively (Supplementary Table 2). TLE-IAS patients with MRI-positive exhibited hypermetabolism in the bilateral frontoparietal regions, the cingulate gyrus, and hypometabolism in the bilateral temporal lobe (*p* < 0.05, FWE corrected). The region of metabolic change in MRI-negative TLE-IAS patients was limited than that in MRI-positive patients. The region of metabolic change in MRI-negative TLE-IAS has included hypermetabolism in the cingulate gyrus and corpus callosum (*p* < 0.05, FWE corrected) (Supplementary Figure 1 and Supplementary Table 2).

## Discussion

Impairment of consciousness during recurrent epileptic seizures has significant consequences on the whole brain function involving perfusion, metabolism, and electrophysiology networks (Blumenfeld, 2012). In this large series of TLE patients with IAS, glucose hypermetabolism was found in most cortical-subcortical regions, especially the bilateral frontoparietal regions, cingulate gyrus, corpus callosum, basal ganglia, thalamus, brainstem, and cerebellum, suggesting that interictal cortico-subcortical metabolic network disturbance might be the results of repetitive consciousness impairing ictal events, contributing to the study of the metabolic mechanism for loss of consciousness in TLE (Blumenfeld, 2012; Calabrò et al., 2015).

Compared to healthy controls, hypometabolism was found in bilateral temporal lobes among TLE-IAS patients, which is probably due to the majority of focal impaired consciousness seizures arising from the temporal lobe or spread to the temporal lobe from other epileptogenic foci (Lee et al., 2002). We also found TLE-IAS patients had widespread hypermetabolism in the bilateral frontoparietal area, supporting the hypothesis that even though focal seizure originates in the focal lobes, it can affect metabolic changes in remote cortical regions. It has long been known that, the orbitofrontal cortex has a high level of functional connectivity with the hippocampus (Engel et al., 1993; Catenoix et al., 2005) and that this connectivity contributes to seizure propagation (Lieb et al., 1991; Engel et al., 1993). Evidence from both neuropsychological and neuroimaging literature suggests that frontal lobe dysfunction is common in focal epilepsy (Bertti et al., 2014; Englot et al., 2018). Due to the role of the frontal lobe in behavioral arousal, attention, and awareness, metabolic disturbances in the neocortex of patients with TLE might be related to consciousness impairment (Blumenfeld et al., 2009; Blumenfeld, 2012; Englot et al., 2018; Satizabal et al., 2019). A previous single-photon emission computed tomography (SPECT) study found that a decrease in cerebral blood flow to the frontoparietal areas during focal temporal lobe seizures correlates with deficits in consciousness (Blumenfeld et al., 2004b). In addition, high amplitude slow oscillations in frontoparietal regions, most prominent in the orbitofrontal cortex, have been recorded on intra- and post-ictal EEG (Guedj et al., 2015; Lamarche et al., 2016; Chibane et al., 2017; Wagner et al., 2017; Roy et al., 2019), suggesting that the decrease in consciousness is due to a depressed cortical state resembling coma or deep sleep (Bagshaw et al., 2014). In our study, widespread metabolic disturbances in the bilateral orbitofrontal cortex of patients with TLE support the theory that the neocortex plays an important role in maintaining normal consciousness.

Several subcortical regions were consistently affected in our cohort of patients with TLE-IAS. Bilateral hypermetabolism was seen in the brainstem, corpus callosum, and cingulate gyrus. Previous work supports the important role that the thalamus and upper brainstem play in attention, arousal, and maintaining consciousness (Blumenfeld, 2012; Calabrò et al., 2015; Koch et al., 2016; Tan et al., 2019). The thalamus is also a principal component of the limbic system and the ascending reticular activating system, which has distinct connections with the cingulate gyrus, reticular formation, and cerebral cortex (Feng et al., 2017). Electrophysiological and neuroimaging studies have shown that seizures can inhibit neural activity and decrease blood flow to the brainstem and thalamus, resulting in cortical dysfunction (Blumenfeld et al., 2009). Conversely, ictal high-frequency electrical stimulation of both the thalamus and brainstem in rodent models can convert cortical slow oscillations to awake fast waves and restore behavioral arousal (Koch et al., 2016; Gutfreund, 2017). In our study, Through the FDR correction method, it is found that the hypermetabolic areas include the basal ganglia, thalamus, and brainstem, which are not involved through the FWE correction methods. Considering that the standard of the FWE correction method is stricter than FDR correction. Hypermetabolism of the basal ganglia, thalamus, and brainstem region are clear, but the degree may not be as high as that cortical and subcortical areas. A study of resting-state fMRI showed widespread increases in local functional connectivity between the midbrain, thalamus, prefrontal cortex, and cerebellar plexus in patients with TLE-IAS (Li et al., 2020). Therefore, metabolism changes in the thalamus and brainstem in our study may provide more evidence to support the view that subcortical arousal system disturbance was responsible for loss of consciousness in TLE.

Of other subcortical structures, the corpus callosum has been shown to play an important role in the regulation of seizures and contribute to seizure propagation. We observed corpus callosum involvement on PET imaging in TLE-IAS patients, compatible with abnormally increased activity in the thalamic pathway. This further supports the hypothesis that focal consciousness impairing seizures can trigger a series of subcortical metabolic disturbances and might contribute to seizure generation and propagation, as well as disorders of consciousness (McDonald et al., 2017). The cingulate gyrus is a principal component of the limbic system. Its anterior and posterior portions possess different thalamic and cortical connections, exhibit different cytoarchitectures, and serve distinct functions. Previous research showed that the anterior and posterior cingulate gyri are widely involved in the maintenance of the conscious state and play a role in complex cognitive and attentional processing (Calabrò et al., 2015). Numerous anatomical connections are found between the medial parietal region (precuneus) or posterior cingulate and the medial prefrontal region (medial frontal region) or anterior cingulate, regions that are functionally integrated into reflective self-awareness and the resting conscious state (Koch et al., 2016). These data suggest that repetitive focal seizures can lead to abnormal cerebral metabolism in subcortical midline networks, especially those involving the corpus callosum, and produce impaired consciousness in TLE.

Consistent with prior SPECT and functional MRI studies (Blumenfeld et al., 2004a; Li et al., 2017), we observed marked hypermetabolism in the bilateral cerebellar hemispheres. Patients with different types of focal epilepsy or generalized epilepsy demonstrate widespread cerebellar hyperperfusion or activation, suggesting a role for the cerebellum in reflective protection and seizure termination during the ictal and postictal periods. Animal studies have shown that cerebellar stimulation can shorten hippocampal epileptiform activity and decrease seizure frequency (Chassoux et al., 2016; Schmahmann, 2019). Since cerebellar Purkinje neurons have strong inhibitory outputs to the thalamus and cortex, it is plausible that cerebellar activation may restore seizure-related cerebral imbalance between inhibition and excitation.

MRI is routinely used to detect structural changes associated with epilepsy and can be used to locate epileptic foci (Woermann and Vollmar, 2009; Muhlhofer et al., 2017). However, thirty percent of patients with TLE are MRI negative (Muhlhofer et al., 2017). In our study, 28 patients with TLE-IAS are absent from visible epileptogenic lesions in MRI examination. We found bilateral temporal lobe hypometabolism in MRI- positive TLE-IAS patients. In addition, our study also found metabolic changes in the cortex-subcortical areas. This means that this metabolic abnormality may go beyond the metabolic abnormality of the MRI-positive TLE itself and involve cortical to subcortical areas. However, only subcortical areas of hypermetabolism were found in MRI-negative patients, but no metabolic abnormalities were found in the temporal lobe. This may be because we did not separate the left and right TLE-IAS, resulting in a metabolic offset. Regardless of MRI-positive and negative, cortical and subcortical brain regions related to TLE-IAS were found. A previous study based on MRI spectroscopy also found that metabolic changes in the brainstem and thalamus were observed in patients with epilepsy with impaired consciousness (Tan et al., 2019). Therefore, it is still necessary to further study the difference between MRI and PET in TLE-IAS patients.

Different seizures that cause consciousness impairment converge on the same set of neuroanatomical structures, yet not all seizures affect consciousness through the same mechanism. Thus, the key to unlocking the mechanism of consciousness impairment lies not just in “where,” but in “how,” in other words, the pathophysiology of how these neuroanatomical regions interact to form a widespread brain network disturbance. It is traditionally believed that neuronal dysfunction, hypometabolism, and hypoperfusion of cortico-subcortical structures are responsible for seizure-related impairment of consciousness (Blumenfeld, 2012; Stender et al., 2014; Li et al., 2019; Tan et al., 2019). In the present study, we showed interictal glucose hypermetabolism in cortico-subcortical regions available in TLE patients and speculated it was associated with awareness impairing seizures. Interictal PET is thought to reflect glucose uptake of neurons and glial cells in the whole brain, and local or widespread glucose hypermetabolism represents neuroinflammatory microglia proliferation or activity during interictal periods (Brabazon et al., 2017; Liu et al., 2017; Dienel et al., 2018). This offers a possible pathophysiological mechanism for seizure-related cognition deficits: consciousness-impairing focal seizures recur, which stimulates microglia proliferation in cortico-subcortical structures, leading to constant neuroinflammation and eventually results in hypermetabolism (Nehlig and Coles, 2007; Gershen et al., 2015). Furthermore, large areas of hypermetabolism in the contralateral subcortical and subcortical regions seem to be due to the restoration of chemical homeostasis (Franceschi et al., 1995). Meanwhile, cerebellar activation is triggered to restore seizure-related cerebral imbalance, leading to cerebellar hypermetabolism. Lasting cortico-subcortical neuroinflammation aggravates neuron apoptosis and cerebral atrophy, which also explains why thalamic and cerebellar atrophy is common in patients with refractory focal epilepsy.

Several limitations of the present study should be mentioned. Antiseizure medications may have some effect on brain metabolism which should be considered in future studies. In addition, almost all patients with TLE have seizures of disturbance of consciousness, so it is hard for us to find TLE patients with aware seizures as a comparison, which may limit the statistical power of uncovering metabolic network alterations in these patients. To address this concern, we introduced 60 age-matched healthy controls to analyze baseline glucose uptake. We also analyzed the right and left TLE groups separately to detect laterality effects and to ensure the validity of our results. The group of patients with TLE with focal aware seizures can also aid in the further analysis of the awareness-related brain metabolic mechanisms. Our group will continue to accumulate PET data from the focal epileptic patients with TLE, hopefully, we would explore more interesting findings regarding consciousness-impairing metabolic mechanisms in patients with focal seizures in the upcoming future. Additionally, it is not uncommon for patients to present seizures both with or without alteration of consciousness, evaluation using video EEG, or epilepsy history. An accurate and objective assessment of consciousness during epileptic seizures is needed.

In summary, our study was the first meaningful attempt to investigate possible metabolic mechanisms for impairment of awareness in patients with TLE. We demonstrated that the patients with focal impaired awareness seizures exhibited metabolic alterations in more extensive subcortical systems and cortical networks. Our findings suggest that the repetitive consciousness impairing ictal events may have an accumulative effect on brain metabolism, resulting in abnormal interictal cortical-subcortical metabolic disturbance in focal impaired awareness seizures. Understanding these metabolic mechanisms could greatly guide future clinical treatments to prevent consciousness and cognition impairment and improve the quality of life among people with epilepsy.

## Data Availability Statement

To facilitate full transparency and openness of data, the dataset used and analyzed for the current study is available from the corresponding author on reasonable request.

## Ethics Statement

The studies involving human participants were reviewed and approved by Ethical Commission of Medical Research Involving Human Subjects at Region of Xiangya Hospital, Central South University, China [No. (201412455)]. Written informed consent to participate in this study was provided by the participants’ legal guardian/next of kin.

## Author Contributions

JH designed the method, acquisition of data, and prepared the manuscript. LF and YT designed the method, aided in data analysis, and revised and approved the manuscript. HZ, LX, CZ, and GL aided in data acquisition and interpretation. All authors contributed to the article and approved the submitted version.

## Conflict of Interest

The authors declare that the research was conducted in the absence of any commercial or financial relationships that could be construed as a potential conflict of interest.

## Publisher’s Note

All claims expressed in this article are solely those of the authors and do not necessarily represent those of their affiliated organizations, or those of the publisher, the editors and the reviewers. Any product that may be evaluated in this article, or claim that may be made by its manufacturer, is not guaranteed or endorsed by the publisher.

## Footnotes

1
http://www.alivelearn.net/xjview
