# Supplementary material for: Alterations in Cortical-Subcortical Metabolism in Temporal Lobe Epilepsy With Impaired Awareness Seizures
Source: Front Aging Neurosci. 2022 Mar 10;14:849774. doi: 10.3389/fnagi.2022.849774 (PMC8961434; doi:10.3389/fnagi.2022.849774)
Supplement: Supplementary file 1 [file Data_Sheet_1.pdf]

Supplementary material files

Article title: Alterations in cortical-subcortical metabolism in temporal lobe epilepsy with impaired awareness seizures

Supplementary Table 1: Demographics of MRI-positive and MRI-negative patients**.**

	**MRI-positive**	**MRI-negative**	**P value**
**Age (years)**	27.8 (10.6)	22.8 (8.6)	0.037*
**Sex (male: female)**	22:26	19:9	0.063
**Onset age of seizure (years)**	12.0 (8.4)	8.9 (6.0)	0.058
**Duration of seizure (years)**	17.0 (10.9)	13.9 (9.2)	0.210
**SEEG**	7	5	
**Postoperative pathology**			
FCD	9	1	
HS	15	4	
Tumor	2	0	
other	10	7	
**Epileptogenic zone**			
Left temporal lobe	23	18	
Right temporal lobe	25	10	

*:P<0.05

Abbreviations: FCD, focal cortical dysplasia; TLE−IAS, temporal lobe epilepsy with impaired awareness seizures; HS, hippocampal sclerosis; SEEG, stereo-electroencephalography.

Suppelmentary Table 2: Location and peaks of significant reduction/increasing in glucose metabolism in TLE-IAS patients with MRI-positive and MRI-negative compared with normal controls, repectively.

Cluster-level k_E	Voxel-level T	p $_{FWE\text{-}corrected}$	Peak coordinates (x, y, z) (mm)			Anatomical region	Brodmann area
**MRI positive vs control**							
1876	7.71	0.000	-14	-4	42	Left cingulate gyrus	
	7.45	0.000	-14	44	-2	Left anterior cingulate	32
	7.35	0.000	-22	-28	44	Left frontal lobe/ sub-gyral	
1662	7.55	0.000	16	20	30	Right cingulate gyrus	
	6.47	0.000	18	38	14	Right anterior cingulate	
	6.45	0.000	20	-22	46	Right frontal lobe/ sub-gyral	
39	5.79	0.001	-34	-42	30	Left parietal lobe/ sub-gyral	
49	5.37	0.004	22	16	16	Right sub-lobar	
33	5.32	0.005	-26	-70	16	Left temporal lobe/ sub-gyral	
43	5.13	0.009	38	-60	-2	Right temporal lobe/ sub-gyral	
427	-6.08	0.000	44	14	-36	Right middle temporal gyrus	
	-6.03	0.000	58	4	-16	Right middle temporal gyrus	
	-6.01	0.000	50	-14	-36	Right inferior temporal gyrus	
32	-4.97	0.017	64	-14	-6	Right middle temporal gyrus	
	-4.73	0.040	66	-20	-12	Right middle temporal gyrus	21
**MRI negative vs control**							
35	5.40	0.005	-20	-28	40	Left cingulate gyrus	
37	5.23	0.009	34	-30	-8	Right sub-lobar	
33	5.23	0.009	-12	26	20	Left corpus callosum	
20	5.17	0.011	-14	-6	38	Left cingulate gyrus	
30	4.87	0.030	4	20	18	Right corpus callosum	
	4.85	0.033	6	12	24	Right corpus callosum	

Supplementary Fig 1: Metabolic features in patients with MRI-positive and MRI-negative temporal lobe epilepsy with impaired awareness seizures (FWE corrected).

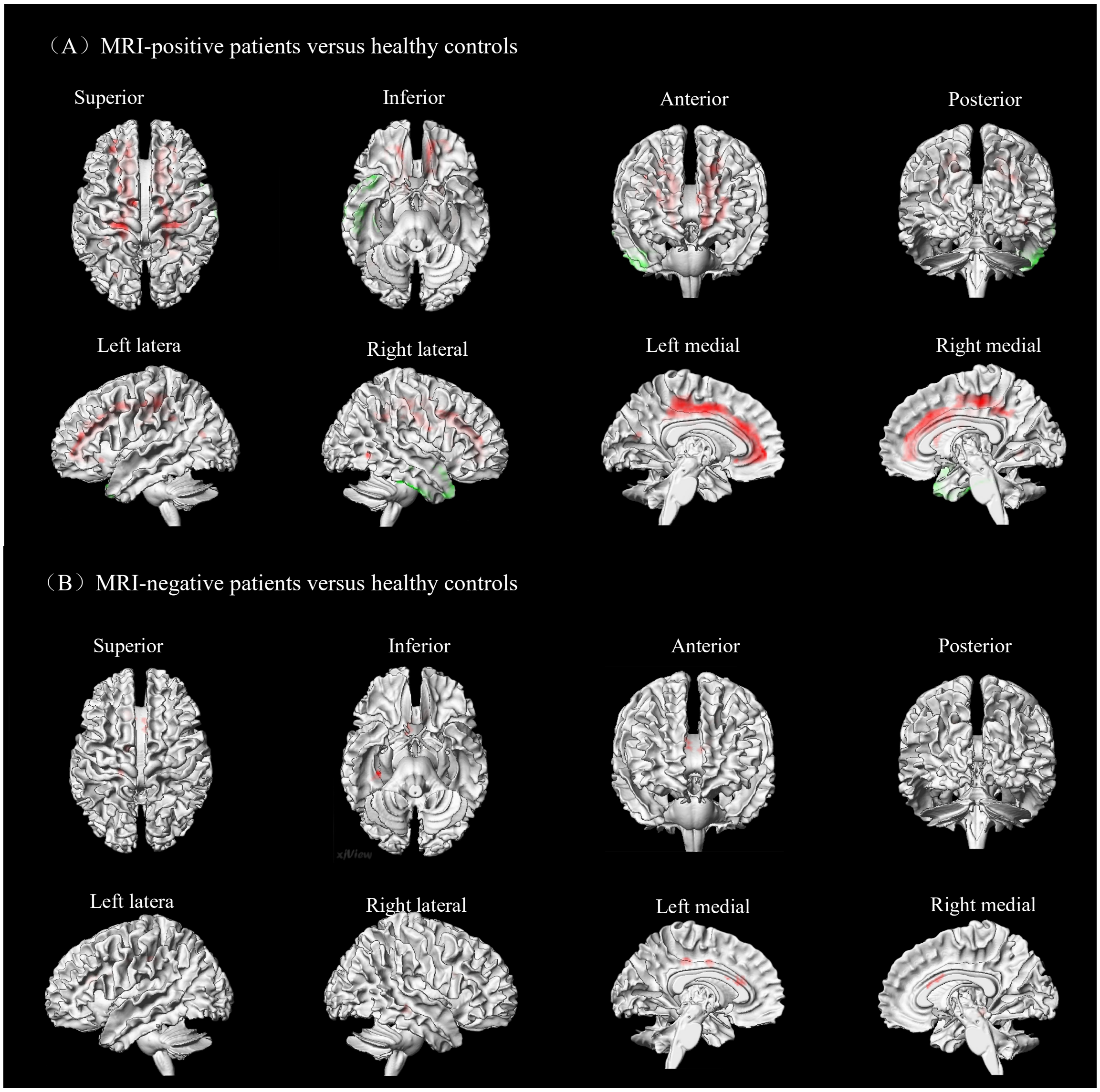

Low uptake are shown in green and high uptake are shown in red. (A) MRI-positive versus healthy controls, (B) MRI-negative patients versus healthy controls. TLE-IAS patients with MRI-positive exhibited hypermetabolism in the bilateral frontoparietal regions, the cingulate gyrus, and hypometabolism in the bilateral temporal lobe. The region of metabolic change in MRI-negative TLE-IAS patients has included hypermetabolism in the cingulate gyrus and corpus callosum. TLE-IAS, temporal lobe epilepsy with impaired awareness seizures.
